# Interleukin-10 produced by myeloid-derived suppressor cells is critical for the induction of Tregs and attenuation of rheumatoid inflammation in mice

**DOI:** 10.1038/s41598-018-21856-2

**Published:** 2018-02-28

**Authors:** Min-Jung Park, Sung-Hee Lee, Eun-Kyung Kim, Eun-Jung Lee, Jin-Ah Baek, Sung-Hwan Park, Seung-Ki Kwok, Mi-La Cho

**Affiliations:** 1 The Rheumatism Research Center, The Catholic University of Korea, Seoul, South Korea; 2 Division of Rheumatology, Department of Internal Medicine, Seoul St. Mary’s Hospital, College of Medicine, The Catholic University of Korea, Seoul, South Korea

## Abstract

Myeloid-derived suppressor cells (MDSCs) are heterogenous populations of immature myeloid progenitor cells with immunoregulatory function. MDSCs play critical roles in controlling the processes of autoimmunity but their roles in rheumatoid arthritis (RA) are controversial. The present study was undertaken to investigate whether MDSCs have therapeutic impact in mice with collagen-induced arthritis (CIA), an animal model of RA. We also examined the mechanisms underlying the anti-arthritic effect of MDSCs. *In vitro* treatment with MDSCs repressed IL-17 but increased FOXP3 in CD4+ T cells in mice. *In vivo* infusion of MDSCs markedly ameliorated inflammatory arthritis. Th17 cells and Th1 cells were decreased while Tregs were increased in the spleens of MDSCs-treated mice. MDSCs profoundly inhibited T cell proliferation. Addition of anti-IL-10 almost completely blocked the anti-proliferative effects of MDSCs on T cells. Anti-IL-10 blocked the expansion of Tregs by MDSCs. However, infusion of MDSCs from IL-10 KO mice failed to suppress inflammatory arthritis. MDSCs could reciprocally regulate Th17/Treg cells and suppress CIA via IL-10, suggesting that MDSCs might be a promising therapeutic strategy for T cell mediated autoimmune diseases including RA.

## Introduction

Myeloid-derived suppressor cells (MDSCs) are a heterogenous group of immune cells from the myeloid lineage. MDSCs strongly expanded under pathologic conditions including the tumor environment and chronic inflammation. They play a pivotal role owing to their potent suppressive activities in immune response^1,2^. These cells produce immunoregulatory mediators including arginase-1, inducible nitric oxide synthase (iNOS), and reactive oxygen species (ROS), which can inhibit the activation of various immune cells, especially T cells^3^. Murine MDSCs can be characterized by the expression of CD11b and Gr-1. As Gr-1+ cells are composed of monocytic and granulocytic cells, murine MDSCs are now divided into two subset; monocytic MDSCs (M-MDSC), defined as CD11b+ Ly6G-Ly6C^high^ cells and granulocytic MDSCs (G-MDSC), defined as CD11b+ Ly6G+ Ly6C^low^ cells^3,4^.

Rheumatoid arthritis (RA) is a prototype systemic autoimmune disease that is characterized by a hyperplastic synovial membrane capable of destroying adjacent articular cartilage and bone^5,6^. Although the pathogenesis of RA has not been fully elucidated, it is certain that T cells are critically implicated in the pathogenesis of RA^7^. A variety of biologic agents targeting proinflammatory cytokines such as TNF-α and IL-6 have proved to be superior to conventional disease-modifying antirheumatic drugs (DMARDs)^8–11^. However, some RA patients are still refractory to biologic agents as well as DMARDs. Therefore, new therapeutic strategies for RA need to be developed.

Considering the potent immunoregulatory effect of MDSCs on T cells, it can be spec ulated that MDSCs might have therapeutic effect on RA. As expected, some reports have demonstrated that adoptive transfer of MDSCs have therapeutic effects in animal model of RA^12–16^. However, a few recent papers have shown that MDSCs can aggravate inflammatory arthritis in mice^17–19^. Thus, the precise impact of MDSCs on RA remains still unclear.

In this study, we attempted to determine the net effects of MDSCs on RA. To do this, we checked whether *in vivo* infusion of various MDSCs including total MDSCs, G-MDSC, and M-MDSC has therapeutic effect in mice with collagen-induced arthritis (CIA), a prototype animal model of RA. We also examined the effect of MDSCs on various T cell populations, including Th1 cells, Th17 cells, and Tregs both *in vitro* and *in vivo*. Both Th1 cells and Th17 cells are proinflammatory T helper cell subsets that are deeply involved in the development of RA^20^. Tregs can attenuate autoimmune response and maintain peripheral tolerance^21^. The underlying mechanisms of anti-arthritis effect of MDSCs were also determined both *in vitro* and *in vivo*.

## Results

### Myeloid-derived suppressor cells (MDSCs) derived from CIA mice decrease IL-17 but increase FOXP3 in CD4+ T cells *in vitro*

**Figure 1:**
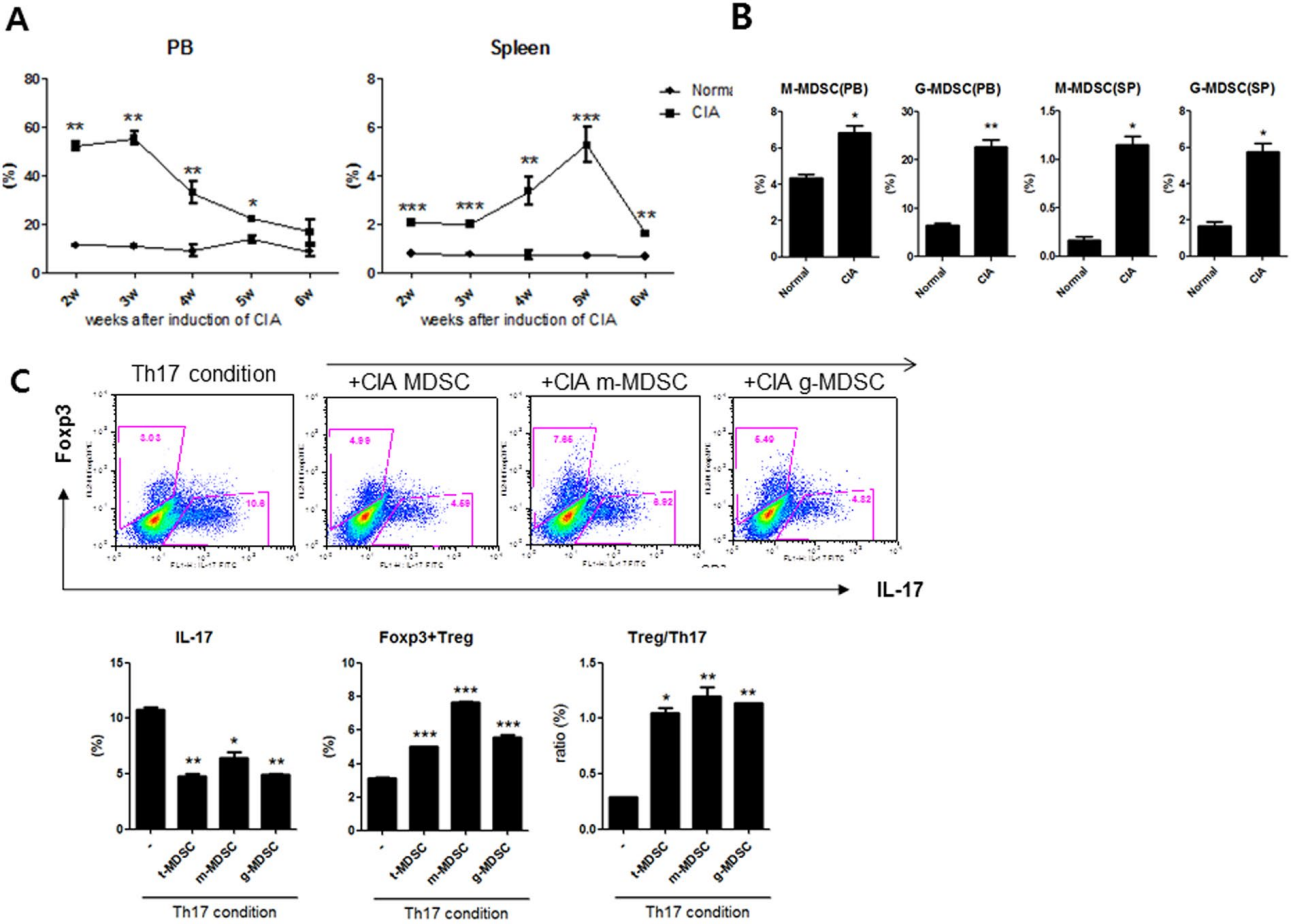
MDSCs repress IL-17 but increase Foxp3 in CD4+ T cells in mice (*in vitro*). (**A**) The percentages of total MDSCs (CD11c-CD11b+ GR-1+ cells) in the spleens and peripheral blood (PB) of CIA mice and control mice (DBA/1 J mice) were analyzed using flow cytometry (n = 6 per group). *P < 0.05, **P < 0.01, ***P < 0.001. (**B**) The percentage of M-MDSCs (CD11c-CD11b+Ly6G-Ly6C^high^ cells) and G-MDSCs (CD11c-CD11b+Ly6G+Ly6C^low^ cells) in spleens and peripheral blood of CIA mice (five weeks after CIA induction). *P < 0.05, **P < 0.01. (**C**) CD4+ T cells from the spleens of CIA mice were cultured under Th17 cells-inducing cytokine conditions as described in the Methods section for 72 hours and then co-cultured with three kinds of MDSCs (at 1:1 ratio) obtained from spleens of CIA mice in the presence of anti-CD3 and anti-CD28. After 3 days, cells were stained with antibodies against IL-17 and Foxp3 using intracellular flow cytometric analysis. Representative results from three independent experiments are shown in the upper panel. The percentages of IL-17 producing CD4+ T cells and Foxp3+ CD4+ T cells was shown in lower panel. *P < 0.05, **P < 0.01, ***P < 0.001.

First, we analyzed the population of MDSCs in spleen (SP) and peripheral blood (PB) in CIA mice. CIA mice were induced as described in the Materials and Methods section. The population of MDSCs in the spleen and peripheral blood in both CIA mice and control mice (DBA/1 J mice) were analyzed using flow cytometry. The total MDSCs were defined as CD11c-CD11b+GR-1+cells. Monocytic MDSCs (M-MDSCs) were defined as CD11c-CD11b+Ly6G-Ly6C^high^ cells. Granulocytic MDSCs (G-MDSCs) were defined as CD11c-CD11b+Ly6G+Ly6C^low^ cells. As shown in Fig. 1A, the percentages of total MDSCs in peripheral blood and spleens of CIA mice were significantly higher at all time compared to those of control mice. MDSCs profoundly accumulated in the spleens of CIA mice on day 35 after CIA induction, consistent with previous report^12^. The percentages of both M-MDSCs and G-MDSCs were also significantly higher in CIA mice on day 35 in both peripheral blood and spleen (Fig. 1B).

There have been some discordant reports regarding the impact of MDSCs on Tregs and Th17 cells. In an attempt to clarify the effect of MDSCs on Tregs and Th17 cells *in vitro*, CD4+T cells from CIA mice were cultured under Th17 differentiation condition for 72 hours and then co-cultured additionally with total MDSCs, M-MDSCs, or G-MDSCs obtained from spleens of CIA mice at a ratio of 1:1 for 72 hours. The results showed that all three kinds of MDSCs increased Foxp3 but decreased IL-17 in CD4+T cells, even after some CD4+T cells were differentiated into Th17 cells (Fig. 1C).

### Cell therapy with MDSCs attenuates rheumatoid inflammation in mice

**Figure 2:**
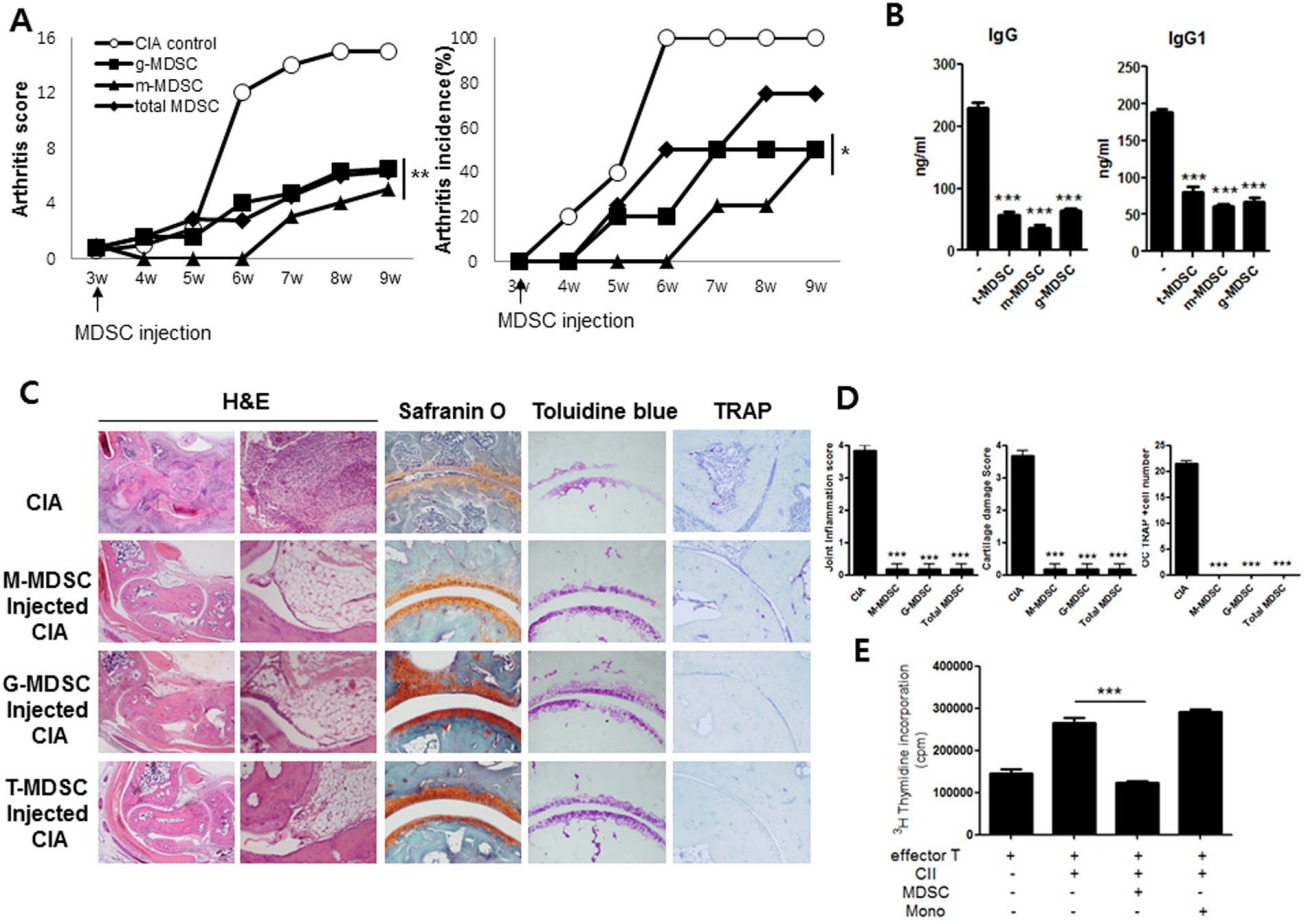
*In vivo* treatment with MDSCs suppresses inflammatory arthritis in mice. (**A**) Reduction in arthritis score and arthritis incidence in CIA mice treated with MDSCs. At three weeks after CIA induction, mice were treated with intravenous infusion of different kinds of MDSCs (5 × 10^5^) (total MDSCs, G-MDSCs, or M-MDSCs) (n = 6 per group). *P < 0.05, **P < 0.01. (**B**) Levels of circulating IgG and IgG1 in CIA mice treated with different kinds of MDSCs. ***P < 0.001. (**C**,**D**) Histologic examinations of the joints of the CIA mice treated with MDSCs. Mice were killed on day 56 after CIA induction. Tissue sections from the joints of each mouse were stained with H&E, safranin O, toluidine blue, and TRAP. Representative photographs from each group are shown in C. The histologic scores of inflammation and cartilage damage as well as the number of osteoclasts are shown in D. ***P < 0.001. (**E**) T cells obtained from spleens of CIA mice were cultured in the presence of 50 *μg*/ml CII with or without MDSCs and/or monocytes from CIA mice for 72 hours. T cell proliferative responses were determined by [^3^H]thymidine incorporation assay. ***P < 0.001.

Next, we tested whether *in vivo* treatment with MDSCs could suppress inflammatory arthritis and joint destruction in CIA mice. On day 21 after induction of CIA, mice were treated with a single intravenous infusion of 5 × 10^5^ MDSCs obtained from spleens of CIA mice. As shown in Fig. 2A, treatment with MDSCs including total MDSCs, G-MDSCs, and M-MDSCs significantly reduced arthritis score and arthritis incidence. Circulating lgG and IgG1 levels were significantly lower in CIA mice treated with MDSCs (Fig. 2B). Histologic examination showed that joints of CIA mice treated with MDSCs exhibited lower degree of inflammation and cartilage damage compared to those of CIA mice without such treatment (Fig. 2C,D). The effects of MDSCs on T cell proliferative response to type II collagen (CII) were also determined. The results showed the addition of MDSCs obtained from CIA mice profoundly decreased T cell proliferative response to CII whereas the addition of monocytes failed to show any impact (Fig. 2E).

### *In vivo* infusion of MDSCs increases Tregs but decreases Th1 and Th17 cells in CIA mice

**Figure 3:**
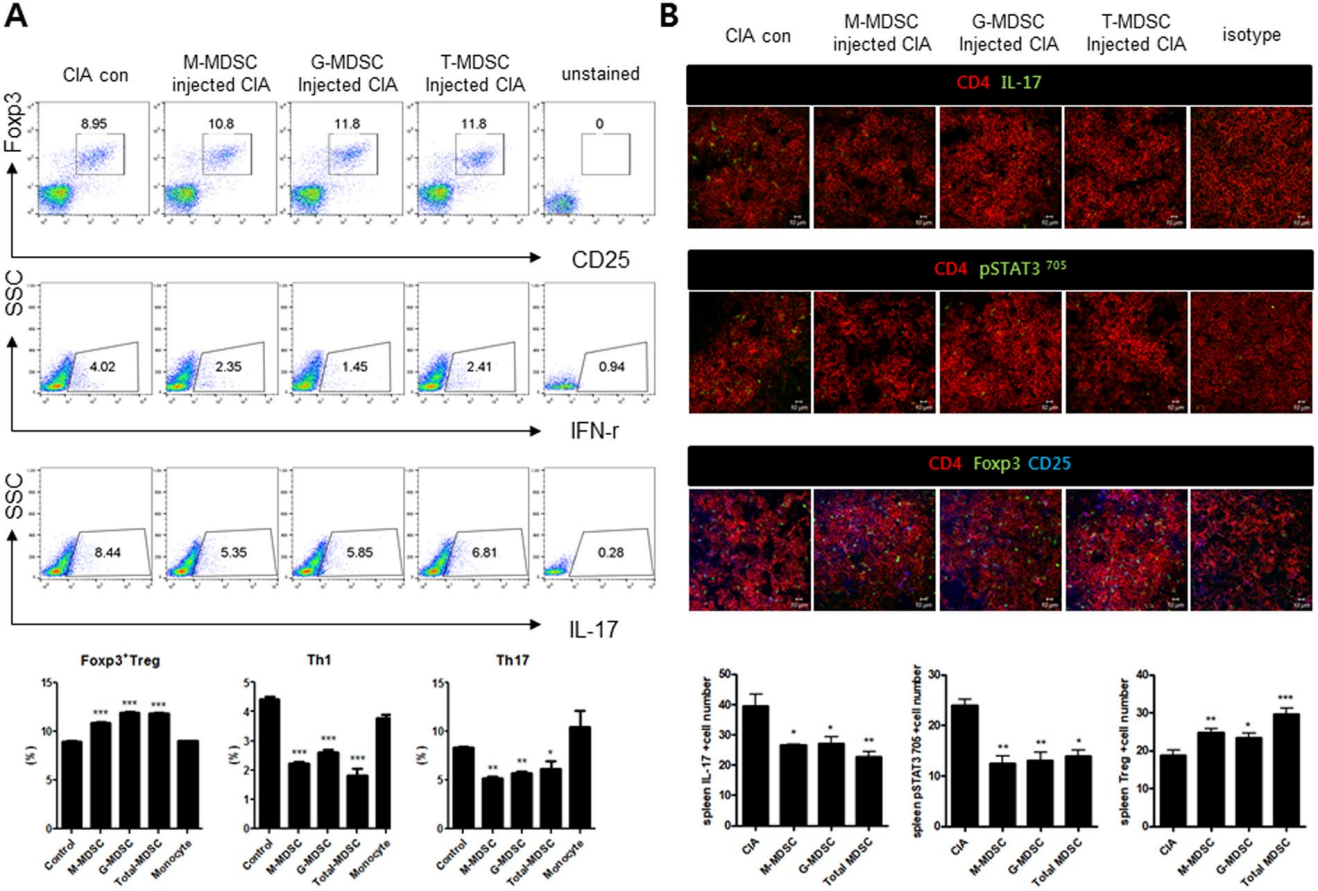
Cell therapy with MDSCs increases Tregs but decreases Th1 and Th17 cells in CIA mice. (**A**) Flow cytometric analysis shows the Tregs (CD4+CD25+FOXP3+), Th1 cells (CD4+IFN-γ+), and Th17 cells (CD4+IL-17+) in spleen tissues of CIA mice treated with different kinds of MDSCs. Representative results from three independent experiments are shown in the upper panel. Relative bar charts are shown in the lower panel. *P < 0.05, **P < 0.01. (**B**) Spleen tissues from each mouse were stained for CD4+ IL17+ T cells, CD4+ phosphoSTAT3+T cells, and CD4+CD25+Foxp3+ T cells using antibodies specific for CD4, IL-17, phosphoSTAT3^705^, CD25, and Foxp3. Theses cell populations were analyzed using laser confocal microscopy (original magnification × 400). The cells showing positive staining for CD4+IL17+ T cell, CD4+ phosphoSTAT3+ T cell, or CD4+ CD25+ Foxp3+ T cells were enumerated visually at higher magnification (projected on a screen) by four individuals, and the mean values are presented in the form of histogram (lower panel). *P < 0.05, **P < 0.01, ***P < 0.001.

We next checked the effect of *in vivo* treatment with MDSCs on various effector T cell subsets. Populations of Tregs, Th1 cells, and Th17 cells in the spleens of CIA mice treated with MDSCs were analyzed with flow cytometry. As shown in Fig. 3A, infusion of MDSCs including total MDSCs, G-MDSCs, and M-MDSCs increased the population of Tregs (CD4+CD25+FOXP3+ cells) in the spleens of CIA mice. However, the populations of CD4+IFN-γ T cells (Th1 cells) and CD4+IL-17+ T cells (Th17 cells) in the spleens were decreased by the infusion of MDSCs.

STAT3 is one of the major transcription factor for the differentiation of Th17 cells^22^. Therefore, we checked the CD4+pSTAT30(Y705)+ T cells as well as CD4+IL-17+ T cells in the spleens of each mice using confocal microscopy. The results revealed that both CD4+pSTAT3 (Y705)+ T cells and CD4+IL-17+ T cells were decreased in CIA mice with *in vivo* treatment with MDSCs. However, Tregs (CD4+CD25+FOXP3+ cells) were increased in the spleens of CIA mice after *in vivo* treatment with MDSCs (Fig. 3B).

### Inhibitory effect of MDSCs on T cell proliferation is mediated by IL-10 and arginase-1

**Figure 4:**
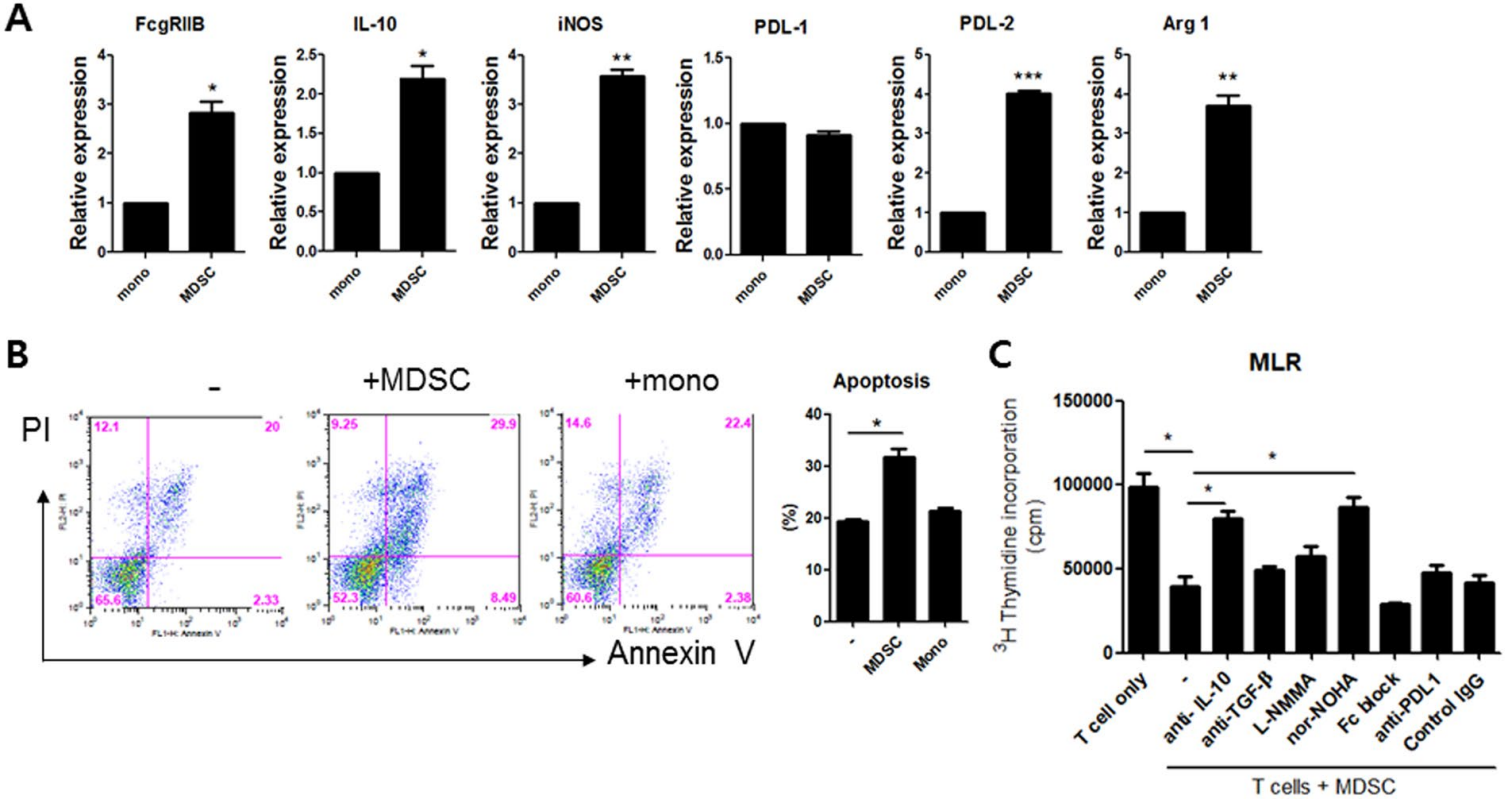
MDSCs inhibit T cell proliferation via IL-10 and arginase-1 *in vitro*. (**A**) Monocytes and MDSCs (CD11c-CD11b+ GR-1+ cells) were obtained from CIA mice at five weeks after induction of CIA. The mRNA levels of various immunoregulatory molecules including FcγRIIB (CD32), IL-10, iNOS, PDL-1, PDL-2, and arginase 1 were measured using real time PCR. *P < 0.05, **P < 0.01, ***P < 0.001. (**B**) T cells isolated from CIA mice were cultured for 72 hours in medium alone, with MDSCs, or monocytes (at 1:1 ratio) obtained from CIA mice as described in Materials and Methods section. The degree of apoptosis was assessed by flow cytometry using propidium iodide (PI) and Annexin V. Apoptotic cells were defined as PI-Annexin V+ cells. Representative results from three independent experiments are shown in the left panel. The percentage of apoptotic T cells is shown in the right panel. *P < 0.05. (**C**) T cells were cultured with MDSCs (at 1:1 ratio) for 72 hours in the presence or absence of anti-IL-10 (10 *μg*/ml), anti-TGF-β (10 *μg*/ml), L-NMMA (500 μM), nor-NOHA (500 μM), anti-FcγRIIB (2 *μg*/ml), or anti-PDL-1 (5 *μg*/ml). T cell proliferative responses were determined by [^3^H]thymidine incorporation assay. *P < 0.05.

It is well-known that MDSCs produce immunosuppressive factors such as arginase-1, inducible nitric oxide synthase (iNOS), or reactive oxygen species (ROS) which can inhibit the activation of various immune cells, especially T cells^3^. In order to find real factors that could mediate the immunomodulatory role of MDSCs, we measured the mRNA levels of immunoregulatory molecules including FcγRIIB (CD32), IL-10, PDL-1, and PDL-2. We also examined mRNA levels of iNOS and arginase-1 in MDSCs obtained from spleens of CIA mice on day 35 after CIA induction. As shown in Fig. 4A, mRNA levels of FcγRIIB, IL-10, iNOS, arginase-1, and PDL-2 in MDSCs were significantly higher than those in monocytes. We also investigated the effect of MDSCs on the apoptosis and/or proliferation of T cells *in vitro*. The results showed that treatment with MDSCs promoted apoptosis of T cells. However, treatment with monocytes failed to have any impact on T cell apoptosis (Fig. 4B). Moreover, treatment with MDSCs significantly inhibited T cell proliferation. Addition of anti-IL-10 or nor-NOHA, an inhibitor of arginase-1, completely blocked the anti-proliferative effects of MDSCs on T cells (Fig. 4C). These findings suggest that MDSCs can inhibit T cell proliferation partly via IL-10 and arginase-1.

### MDSCs expand Tregs via IL-10 *in vitro*

**Figure 5:**
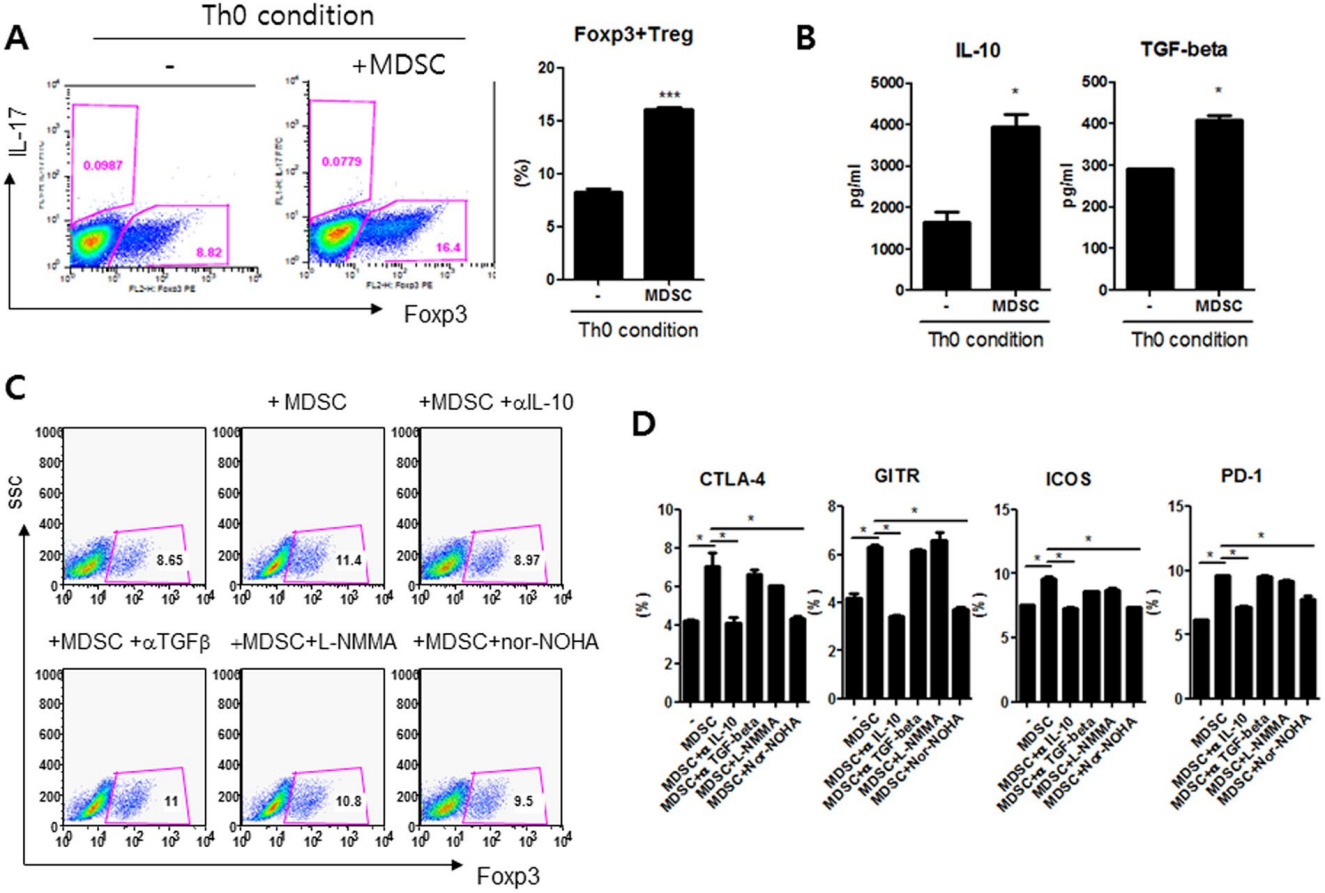
MDSCs expand Tregs via IL-10 *in vitro*. (**A**,**B**) CD4+ T cells obtained from CIA mice were cultured in medium alone or with MDSCs (CD11c-CD11b+GR-1+ cells) (at 1:1 ratio) for 72 hours under Th0 condition (anti-CD3 (0.5 *μg*/ml) and anti-CD28 (0.5 *μg*/ml)). Treg populations (CD4+FOXP3+ cells) were determined by flow cytometry. Representative results from three independent experiments and relative bar charts are shown in A. ***P < 0.001. The levels of TGF-β and IL-10 in culture supernatant were measured by ELISA. *P < 0.05. (**C**,**D**) CD4+ T cells were cultured again with MDSCs (at 1:1 ratio) under Th0 condition for 72 hours in the presence or absence of anti-IL-10 (10 *μg*/ml), anti-TGF-β (10 *μg*/ml), L-NMMA (500 μM), or nor-NOHA (500 μM). The representative flow cytometric contour plots of CD4+FOXP3+ cells were shown in C. The percentage of FOXP3+CTLA-4+, FOXP3+GITR+, FOXP3+ICOS+, or FOXP3+ PD-1+ cells among CD4+ CD25+ T cells were shown in D. *P < 0.05.

We next cultured CD4+ T cells obtained from CIA mice in medium alone or with MDSCs (CD11c-CD11b+GR-1+ cells) (at 1:1 ratio) for 72 hours under Th0 condition (anti-CD3 0.5 *μg*/ml, anti-CD28 0.5 *μg*/ml). As expected, *in vitro* treatment with MDSCs expanded Treg populations (CD4+FOXP3+ cells), which were determined by flow cytometry analysis (Fig. 5A). The levels of IL-10 and TGF-β, two major anti-inflammatory cytokines, were significantly higher in supernatants when CD4+ T cells were co-cultured with MDSCs (Fig. 5B).

In an attempt to unravel the mechanism of Tregs expanding effect of MDSCs, CD4+ T cells were cultured with MDSCs (at 1:1 ratio) under Th0 condition for 72 hours in the presence or absence of anti-IL-10 (10 *μg*/ml), anti-TGF-β (10 *μg*/ml), L-NMMA (500 μM), an iNOS inhibitor, or nor-NOHA (500 μM). Tregs populations and Tregs associated molecules including CTLA-4, GITR, ICOS, and PD-1 were determined using flow cytometry. As shown in Fig. 5A, addition of anti-IL-10 totally blocked the Treg expanding effects of MDSCs. However, addition of anti-TGF-β, L-NMMA, or nor-NOHA failed to have significant effect. Regarding the percentages of FOXP3+CTLA-4+, FOXP3+GITR+, FOXP3+ICOS+, and FOXP3+PD-1+cells among CD4+CD25+T cells, only the addition of anti-IL-10 consistently reverse the effects of MDSCs (Fig. 5D). These findings indicated that the Tregs expanding effect of MDSCs might be mediated by IL-10 to some degree.

### Cell therapy with IL-10 deficient MDSCs does not suppress inflammatory arthritis

**Figure 6:**
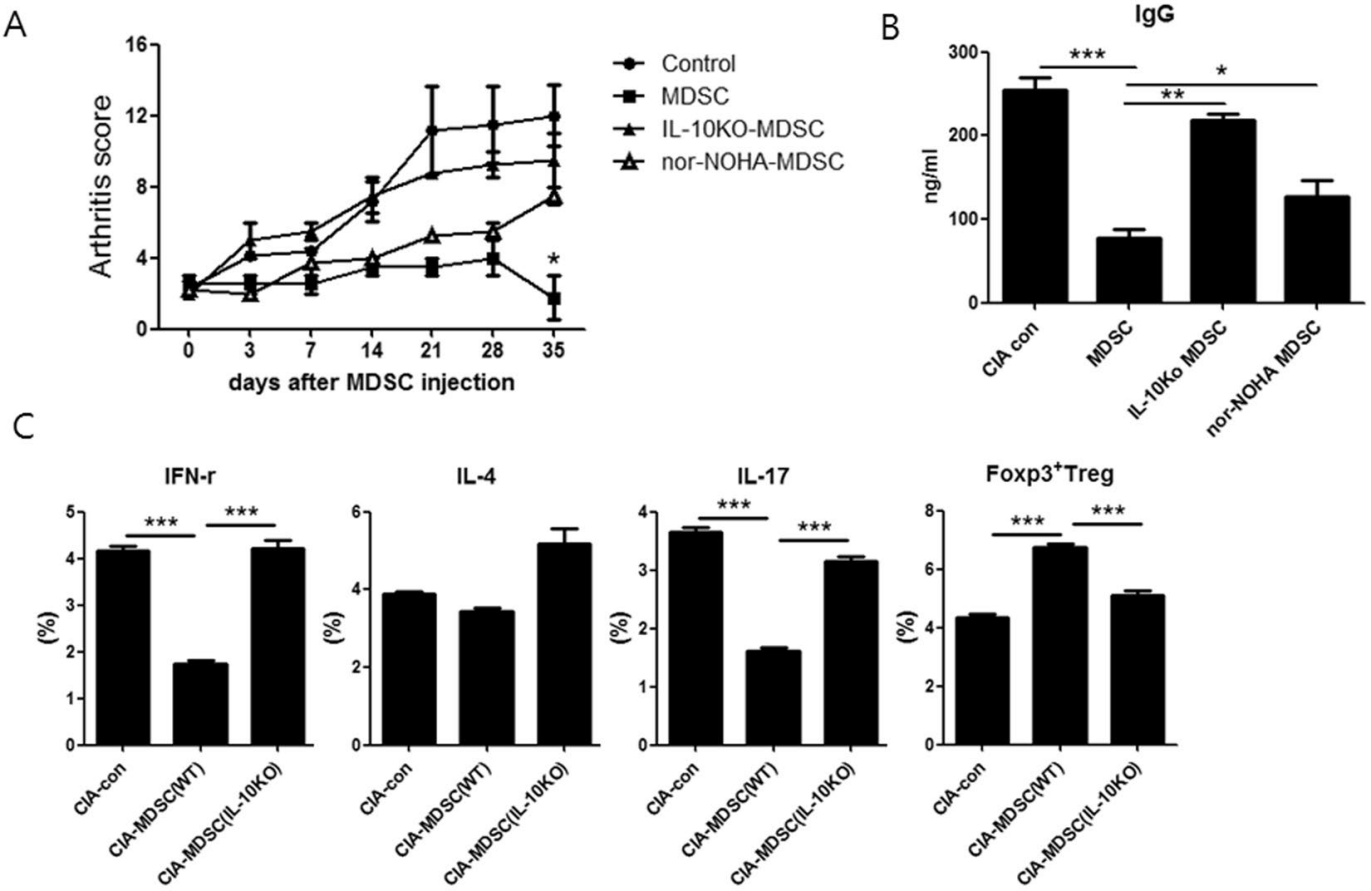
Cell therapy with MDSCs obtained from IL-10 KO mice does not attenuate inflammatory arthritis. (**A**) CIA was induced in DBA1/1 J mice. At three weeks after CIA induction, mice were treated with intravenous infusion of MDSCs (CD11c-CD11b+ GR-1+ cells) (5 × 10^5^) (MDSCs obtained from CIA mice and CIA induced IL-10 KO mice. MDSCs obtained from CIA mice and then pretreated with nor-NOHA MDSCs) (n = 6 per group). Arthritis score was shown in A. *P < 0.05. (**B**) Serum IgG levels in each group of mice were measured using ELISA at eight weeks after CIA induction. *P < 0.05, **P < 0.01, ***P < 0.001. (**C**) Populations of CD4+ IFN-γ+ T cells (Th1 cells), CD4+ IL-4+ T cells (Th2 cells), CD4+ IL-17+ T cells (Th17 cells), and CD4+ FOXP3+ T cells (Tregs) were analyzed in spleen tissues from each group of mice based on flow cytometry analysis. The percentage of IFN-γ+ or IL-4+ or IL-17+ or FOXP3+ cells among CD4+ T cells is shown. ***P < 0.001.

We eventually performed adoptive transfer using MDSCs lacking IL-10 in order to verify that IL-10 produced by MDSCs had a critical role in immunosuppressive activity of MDSCs. The CIA was induced in male DBA1/1 J mice. At three weeks after CIA induction, mice were treated with a single intravenous infusion of MDSCs (CD11c-CD11b+GR-1+cells) (5 × 10^5^). (MDSCs obtained from CIA mice and CIA induced IL-10 KO mice. MDSCs obtained from CIA mice and then pretreated with nor-NOHA) (n = 6 per group). As expected, *in vivo* treatment with MDSCs obtained from CIA mice significantly reduced arthritis score. However, there was no significant difference in arthritis score between CIA mice without treatment (control) and CIA mice treated with MDSCs obtained from CIA induced IL-10KO mice (Fig. 6A). Regarding serum levels of IgG, there was no significant difference between CIA mice without treatment and CIA mice treated with MDSCs obtained from CIA induced IL-10KO mice (Fig. 6B).

We also checked the effector CD4+ T cell subsets in the spleens of mice. As shown in Fig. 6C, infusion of MDSCs derived from CIA mice decreased the population of CD4+IFN-γ T cells (Th1 cells) and CD4+IL-17+ T cells (Th17 cells) but increased the population of CD4+FOXP3+ T cells (Tregs). However, infusion of MDSCs derived from CIA induced IL-10 KO mice failed to have any effect on the population of Th1 cells, Th17 cells, or Tregs.

These findings suggest that IL-10 produced by MDSCs might be critical in the attenuation of rheumatoid inflammation in mice.

## Discussion

The purpose of the study was to investigate whether the myeloid-derived suppressor cells (MDSCs) have therapeutic impact in CIA mice, a representative murine model of RA and to identify the mechanism underlying anti-arthritic effect of MDSCs.

MDSCs are a heterogeneous group of myeloid cells that can suppress immune responses especially in cancer. The majority of published papers on MDSCs derived from the field of cancer research^2^. However, recent studies have demonstrated that these cells have strong immunomodulatory activities in various immune-mediated inflammatory conditions beyond cancer. The mechanisms of MDSC-induced immune suppression have been widely studied. It is well-known that the main immunoregulatory activity of MDSCs is associated with the production of arginase-1, iNOS, and ROS, which can induce the inactivation of various immune cells, especially T cells^1^. Given the potent immuoregulatory activity of MDSCs, it could be expected that MDSCs could have therapeutic impact on RA. There have been discordant results regarding the effect of MDSCs on RA^12–19^. However, all the previous papers performed adoptive transfer of MDSCs in animal model of RA to verify whether the MDSCs had therapeutic effects on RA. However, the types of MDSCs (total MDSCs, G-MDSCs, and M-MDSCs), sources of MDSCs (splenic MDSCs obtained from mice with inflammatory arthritis or MDSCs induced from bone marrow cells of normal mice), and the cell dose of MDSCs used in adoptive transfer were variable among different papers. Therefore, in order to determine the net effect of MDSCs on RA, we performed adoptive transfer using three types of MDSCs (total MDSCs:CD11c-CD11b+GR-1+ cells, M-MDSCs:CD11c-CD11b+ Ly6G-Ly6C^high^ cells, G-MDSCs:CD11c-CD11b+ Ly6G+ Ly6C^low^ cells) in CIA mice. All these MDSCs were obtained from spleens of CIA mice after 35 days of CIA induction. Our results showed that all three kinds of MDSCs significantly reduced the arthritis score and arthritis incidence (Fig. 2A). On histologic examination of the joints, MDSCs treated CIA mice exhibited lower degree of inflammation and cartilage damage (Fig. 2C,D). The population of splenic MDSCs are highly expanded during the course of CIA (Fig. 1A,B), in agreement with a previous report^12^. Therefore, splenic MDSCs expanded in highly inflamed microenvironment of CIA mice might get potent immuoregulatory activity and thereby have therapeutic effects in animal model of RA.

The ability to inhibit immune cell activation is a pivotal characteristics of MDSC. Although MDSC are involved in inhibition of various immune cells including NK and B cells, inhibition of T cells is considered to be the gold standard for evaluation of MDSC function and inhibition of T cell activity appears to be sufficient for designation of cells as MDSC, on condition that they meet the phenotypic criteria^23^. Therefore, we assess the T cell inhibitory capacity of MDSC using both antigen-specific T cells and antigen-nonspecific T cells. In an attempt to test the impact of MDSC on antigen-specific T cell immune response, the effects of MDSCs on T cell proliferative response to type II collagen (CII) were determined. The results indicated that the addition of MDSCs obtained from CIA mice markedly decreased T cell proliferative response to CII whereas the addition of monocytes failed to show any significant impact (Fig. 2E).

There have been some studies that investigated the interaction between MDSCs and different subsets of CD4+ T cells. However, published data on the role of MDSCs in Trges and Th17 cells are inconsistent^4^. It has become confident that interactions between MDSCs and different subsets of CD4+ T cell are not one-directional^3^. Our *in vitro* experiments demonstrated that all three kinds of MDSCs increased Foxp3 but decreased IL-17 in CD4+ T cells, even after some CD4+ T cells already differentiated into Th17 cells (Fig. 1C). *In vivo* infusion of MDSCs decreased the numbers of Th1 cells and Th17 cells, both of which are proinflammatory T helper cell subsets that have been critically involved in the pathogenesis of autoimmune diseases including RA^20^. However, *in vivo* treatment with MDSCs increased the number of Tregs (Fig. 3A,B) in spleens of CIA mice. Tregs can suppress autoimmune process and maintain peripheral tolerance^24^. A recent paper by Guo *et al*. showed that MDSCs obtained from CIA mice promoted Th17 differentiation in a IL-1 dependent manner and therefore have a proinflammaotory role in the pathogenesis of inflammatory arthritis^19^, which is different from our results. Such discrepancy in the results about the role of MDSCs in various effector CD4+ T cell populations might be due to the context-dependent interaction between MDSCs and different subsets of CD4+ T cells. Further extensive studies are required to solve this issue.

For the first time, we demonstrated that IL-10 produced by MDSCs critically could mediate the immuoregulatory effect of MDSCs. It is well accepted that MDSCs can suppress T cell function via soluble mediators such as arginase-1 and iNOS^1,3,25^. Therefore, arginase-1 or iNOS could mediate the immunoregulatory and anti-arthritic effects of MDSCs. In this study, we hypothesized that other factors in addition to arginase-1 and iNOS could mediate the immuoregulatory effect of MDSCs. Therefore, we determined the expression profiles of various anti-inflammatory molecules in MDSCs from CIA mice and compared to those in monocytes. Interestingly, mRNA levels of IL-10 in addition to iNOS, arginase-1 in MDSCs were significantly higher than those in monocytes (Fig. 4A). *In vitro* treatment with MDSCs significantly inhibited T cell proliferation. Addition of anti-IL-10 or nor-NOHA, an inhibitor of arginase-1, completely blocked the anti-proliferative effects of MDSCs on T cells (Fig. 4C). We also demonstrated that *in vitro* Tregs expanding effects of MDSCs could be mediated by IL-10 (Fig. 5C,D). In an attempt to verify whether IL-10 produced by MDSCs had immunoregulatory effects *in vivo*, we performed adoptive transfer using MDSCs lacking IL-10. To our surprise, MDSCs obtained from CIA induced IL-10 KO mice failed to suppress inflammatory arthritis (Fig. 6A). Collectively, these finding suggest that IL-10 produced by MDSCs is very important in the anti-inflammatory and anti-arthritic effects of MDSCs both *in vitro* and *in vivo*.

Collectively, our results demonstrate that MDSCs reciprocally regulate Th17/Treg cells and attenuate inflammatory arthritis via IL-10 in mice. These findings indicate that MDSCs might be used a promising therapeutics for autoimmune diseases including RA.

## Materials and Methods

### Animals

6-week-old–8-week-old male DBA/1 J or IL-10 knockout mice (SLC, Inc., Shizuoka, Japan) were maintained in an SPF environment. All experimental procedures were examined and approved by the Animal Research Ethics Committee of The Catholic University of Korea (2011-0141-01), in conformity with the National Institutes of Health guidelines.

### Induction of arthritis and injection of MDSC

Collagen-induced arthritis (CIA) was induced in DBA1/J mice. Mice were immunized with 100 μg of chicken CII (Chondrex Inc., Redmond, WA, USA) dissolved overnight in 0.1 N acetic acid (4 mg/ml) in complete Freund’s adjuvant (Chondrex Inc). On day 14, a second injection of CII in incomplete Freund’s adjuvant was administered. The immunization was performed intradermally into the base of the tail. CIA mice were injected intravenously with 5 × 10^5^ MDSC or saline on three weeks after 1^st^ immunization.

### MDSC isolation

Total splenocytes from CIA mice (five weeks after immunization) were immunostained with anti-CD11c, anti-CD11b and anti-Gr-1 Ab (BD Biosciences). CD11c-CD11b+ Gr-1+ MDSC subsets were sorted using a FACS Aria II sorter (BD Biosciences). The purity of the sorted MDSC was >98%.

### Clinical scoring and histological assessment of arthritis

Arthritis score was measured visually twice per week based on the appearance of arthritis in the joints and graded according to Williams *et al*.^26^. The joints of each mouse were fixed in 10% formalin, decalcified in 10% EDTA, and embedded in paraffin wax for histological analysis. Hematoxylin-eosin (H&E) stained sections were scored for inflammation, destruction of cartilage, and bone damage according to published criteria^27,28^.

### Mixed lymphocyte reaction (MLR)

Irradiated antigen presenting cells (APC, 1 × 10^5^ cells) were used as stimulators and CD4+ T cells (1 × 10^5^ cells) were used as responders. Bovine type II collagen (50 ug/ml) was treated with or without MDSC for 3 days at 37 °C. All wells were pulsed with 0.5 μCi [^3^H]thymidine (Amersham Pharmacia. Biotech, Little Chalfont, U.K.) in 20 μl RPMI 1640 for 16 h before the termination of culture. Thymidine incorporation was measured using the Beta-counter system (PACKARD, CA, USA).

Specific inhibitors were used to suppress MDSC-derived inhibitory factors in MDSC and autologous T-cell cocultures at titrated concentrations of anti–IL-10, anti–TGF-β, anti– FcyRII,anti–PDL1 mAb or Control IgG mAb (Functional Grade, eBioscience), L-NMMA (iNOS inhibitor, Sigma), nor-NOHA (ARG1 inhibitor, Sigma).

### Apoptosis assay

To determine the apoptosis of CII reactive T cells, CD4+ T cells were co-cultured MDSC or monocyte with or without CII. After three days, apoptotic cells of CD4+ T cells were detected with flow cytometer using an Annexin V-FITC apoptosis detection kit (Bio Vision, Mountain View, CA).

### Flow cytometry

Mononuclear cells were immunostained with various combinations of fluorescently conjugated antibodies to the following: CD11c, Gr-1, CD25, CD4, Foxp3, IL-17, CD44, CD62L, CTLA-4, GITR, PD-1 and ICOS. The cells were also stained intracellularly with antibodies to CTLA-4 (BD Biosciences), IL-17, and Foxp3 (eBioscience, San Diego, CA, USA). Prior to intracellular staining, cells were restimulated for 4 h with phorbol myristate acetate (25 ng/ml) and ionomycin (250 ng/ml) in the presence of GolgiSTOP (BD Biosciences). Intracellular staining was conducted using a kit (eBioscience), following the manufacturer’s protocol. Flow cytometry was performed using a FACSCalibur instrument (BD Biosciences).

### Reverse transcription–polymerase chain reaction analysis

Messenger RNA (mRNA) was extracted using the TRI Reagent (Molecular Research Center, Inc. Cincinnati, OH, USA) according to the manufacturer’s instructions. Complementary DNA was synthesized using a SuperScript Reverse Transcription system (Takara Bio Inc., Otsu, Japan). A LightCycler 2.0 instrument (software version 4.0; Roche Diagnostics, Mannheim, Germany) was used for PCR amplification. All reactions were performed using the LightCycler FastStart DNA Master SYBR Green I mix (Takara Bio Inc.), following the manufacturer’s instructions. The resulting cDNA was amplified by PCR primer using FcgRIIB sense (5′-ATC CAG GTG CTC AAG GAA GA-3′) and antisense (5′-TGC TCC ATT TGA CAC CGA TA-3′) primers, using IL-10 sense (5′-AAG TGA TGC CCC AGG CA-3′) and antisense (5′- TCT CAC CCA GGG AAT TCA AA-3′) primers, using iNOS sense (5′-GAC CAG CTG GGC TGT ACA AAC CTT-3′) and antisense (5′-CAT TGG AAG TGA AGC GTT TCG-3′) primers and using PD-L1 sense (5′-AAA GTC AAT GCC CCA TAC CG-3′) and antisense (5′-TTC TCT TCC CAC TCA CGG GT-3′) primers, using PD-L2 sense (5′-TGA GGA GCT GTG CTG GGT G -3′) and antisense (5′-CAC ACT GCT GCC CAC GTC TA-3′) primers and using Arginase-1 sense (5′-CAG AAG AAT GGA AGA GTC AG-3′) and antisense (5′-CAG ATA TGC AGG GAG TCA CC-3′) primers. All mRNA levels were normalized to that of β-actin.

### Measurement of cytokine and IgG levels

The concentrations of IL-10 and TGF-β in culture supernatants and serum samples were measured using a sandwich enzyme-linked immunosorbent assay (ELISA) (DuoSet; R&D Systems). Serum levels of IgG and IgG1 antibodies were measured using a commercially available ELISA kit (Bethyl Laboratories, Montgomery, TX, USA).

### Confocal microscopy and immunostaining

Tissues were obtained 42 days after CII immunization, snap-frozen in liquid nitrogen, and stored at −80 °C. Tissue cryosections (7 μm thick) were fixed in 4% (v/v) paraformaldehyde and stained using fluorescein isothiocyanate (FITC)-, phycoerythrin (PE)-, PerCP–Cy5.5-, or allophycocyanin (APC)-conjugated monoclonal antibodies to CD4, CD25, IL-17, Foxp3 and pSTAT-3 (Y705)(all from eBioscience, San Diego, CA, USA). After overnight incubation at 4 °C, the stained sections were visualized by confocal microscopy (LSM 510 Meta; Zeiss, Göttingen, Germany).

### Statistical analysis

Experiments were independently replicated at least twice, and representative and/or summary data are shown. Variation in sample distribution was examined by Shapiro-Wilk test. The experimental values are presented as mean ± standard deviation (SD). Comparisons of numerical data between two groups were performed with Student’s *t*-tests or Mann-Whitney U-test. Differences in the mean values of various groups were analyzed using analysis of variance (ANOVA) with post-hoc test. *P* values less than 0.05 (two-tailed) were considered as statistically significant. All statistical analyses were performed using SAS software (version 9.2; SAS Institute, Cary, NC, USA).
